# Assessment of the relative success of sporozoite inoculations in individuals exposed to moderate seasonal transmission

**DOI:** 10.1186/1475-2875-8-161

**Published:** 2009-07-15

**Authors:** Adama Tall, Cheikh Sokhna, Ronald Perraut, Didier Fontenille, Laurence Marrama, Alioune B Ly, Fatoumata D Sarr, Aïssatou Toure, Jean-François Trape, André Spiegel, Christophe Rogier, Pierre Druilhe

**Affiliations:** 1 Unité d'Epidémiologie, Institut Pasteur de Dakar, B.P. 220 Dakar, Sénégal; 2 UR 077 Paludologie Afrotropicale BP 1386, Dakar, Senegal; 3 URBEP, UMR 6236 – URMITE, IRBA, BP46, BP60109, 13262 Marseille, France; 4 Bio-Medical parasitology Unit, Institut Pasteur de Paris, 28 rue Dr Roux, 75015, France

## Abstract

**Background:**

The time necessary for malaria parasite to re-appear in the blood following treatment (re-infection time) is an indirect method for evaluating the immune defences operating against pre-erythrocytic and early erythrocytic malaria stages. Few longitudinal data are available in populations in whom malaria transmission level had also been measured.

**Methods:**

One hundred and ten individuals from the village of Ndiop (Senegal), aged between one and 72 years, were cured of malaria by quinine (25 mg/day oral Quinimax™ in three equal daily doses, for seven days). Thereafter, thick blood films were examined to detect the reappearance of *Plasmodium falciparum *every week, for 11 weeks after treatment. Malaria transmission was simultaneously measured weekly by night collection of biting mosquitoes.

**Results:**

Malaria transmission was on average 15.3 infective bites per person during the 77 days follow up. The median reappearance time for the whole study population was 46.8 days, whereas individuals would have received an average one infective bite every 5 days. At the end of the follow-up, after 77 days, 103 of the 110 individuals (93.6%; CI 95% [89.0–98.2]) had been re-infected with *P. falciparum*. The median reappearance time ('re-positivation') was longer in subjects with patent parasitaemia at enrolment than in parasitologically-negative individuals (58 days vs. 45.9; p = 0.03) and in adults > 30 years than in younger subjects (58.6 days vs. 42.7; p = 0.0002). In a multivariate Cox PH model controlling for the sickle cell trait, G6PD deficiency and the type of habitat, the presence of parasitaemia at enrolment and age ≥ 30 years were independently predictive of a reduced risk of re-infection (PH = 0.5 [95% CI: 0.3–0.9] and 0.4; [95% CI: 0.2–0.6] respectively).

**Conclusion:**

Results indicate the existence of a substantial resistance to sporozoites inoculations, but which was ultimately overcome in almost every individual after 2 1/2 months of natural challenges. Such a study design and the results obtained suggest that, despite a small sample size, this approach can contribute to assess the impact of intervention methods, such as the efficacy vector-control measures or of malaria pre-erythrocytic stages vaccines.

## Background

Exposure to parasites induces immune responses, which can reduce parasite loads. The acquisition of a state of protection against clinical malaria, called premunition, by individuals who are regularly infected with *Plasmodium falciparum *enables them to control parasite densities to low levels and, thereby, to reduce the incidence of clinical malaria episodes. This control is usually considered to be mainly induced by and effective against erythrocytic forms of *P. falciparum *[1]. However, it has been proposed that the protection acquired by exposure to infection, may also extend to other stages including pre-erythrocytic forms [2]. Experimental studies have shown that exposure to irradiated sporozoites induces parasitological and clinical immunity[2]. In natural conditions, exposure to infected mosquitoe bites induces immune responses to sporozoite surface antigens and their intensity is a function of the Entomological Inoculation Rate (EIR), and, obviously, of age, reflecting the cumulative number of sporozoites received [3-6]. Naturally acquired antibodies also strongly inhibit sporozoite invasion into hepatocytes under in vitro conditions [7].

This exposure-induced immunity may explain why in many hyperendemic areas the incidence of malaria attacks is always markedly lower than predicted by the number of sporozoite inoculations. For instance, 1–5 malaria attacks/child/year are observed in areas where children receive >100 infective inocula by mosquitoes per year [8,9], suggesting a reduction of the proportion of inoculum that leads to a blood parasitaemia proportional to the antigenic natural stimulation by pre-erythrocytic stages of the parasites. However, as the evaluation of immunity is based on the detection of blood parasitaemia, it is difficult in observational studies to distinguish the respective effects of immune responses against pre-erythrocytic and erythrocytic stages. Several studies have attempted to document the existence of a pre-erythrocytic "brake" [9], which may result from immunity acquired by exposure to antigens from sporozoites and liver stages. It has been shown for instance that a tenfold decrease in malaria transmission is associated, with only a two-fold decrease in malaria morbidity [9] and little changes in parasitaemia. In the Garki project, it proved impossible to make a transmission model fit field observations, unless a strong density-dependent pre-erythrocytic filter, or brake, was assumed [8].

One way of quantifying this control of new malarial inoculations, is to measure the time of reappearance of blood parasites in subjects whose parasitaemia has been cleared by radical cure and who are exposed to measured numbers of infective mosquitoe bites (called '*re-positivation*' here), i.e. to measure the relative success of new sporozoite inoculations [8,10-12]. This method, which is recommended by WHO in the field to test the efficacy of candidate vaccines, has been previously used [13].

In the present study, the relationships between the re-infection time and several host factors were analysed in a Senegalese community living in the village of Ndiop, an area of seasonal malaria transmission. The aim was to identify the host factors involved in this immune resistance or the factors that could serve as intermediate criteria for the evaluation of intervention methods.

## Methods

### Follow-up procedure

The existing set-up of Ndiop, a village of about 350 inhabitants, located in the Sine-Saloum, in the sahelo-sudanian region of Senegal, was used. In this village, numerous malaria studies, particularly extremely close monitoring of clinical malaria attacks (daily active case detection), drug use and entomological follow-up have been in place since 1993, according a protocol that was identical to that used in the village of Dielmo, Senegal. Thick blood smears were prepared Giemsa-stained, and examined by 1,000× light microscopy using oil immersion. Parasitaemias were scored per microliter of blood by counting the number of asexual parasites per 100 white blood cells, assuming 8,000 blood cells per microliter, and multiplying the parasite count by 80 [14]. Malaria transmission is seasonal from July to October. Malaria vectors are *Anopheles arabiensis*, *Anopheles gambiae *and *Anopheles funestus *[15]. Biological tests, including haemoglobin electrophoresis and tests for glucose-6-phosphate dehydrogenase (G6PD) deficiency, were available for most subjects participating in the study. The type of habitat and bed net use were recorded for each subject. One hundred eighteen volunteers, aged one year or above, were enrolled in the study at the end of the dry season (in August, 1997). During the study period, treatment relied on the administration of quinine chlorhydrate (Quinimax^®^, Sanofi-Synthelabo, France), chosen in view of its efficacy in this area of chloroquine resistance, of its fast effect, and its short half-life. It was administered orally at a dose of 8 mg/kg under medical supervision (with assessment of proper ingestion) at 8 h intervals (i.e., 25 mg/kg/d) for 7 d. The very short half-life of quinine has the advantage to avoid the persistence of prophylactic concentrations of the drug in the receivers' blood that could provide artificial protection in the following weeks or months, i.e., avoid introducing a confounding factor as compared to other antimalarial drugs. Since free medical care is available 24 h/d, self-treatment is known to be most uncommon, as ascertained by systematic detection of anti-malarial drugs [16]. In case of systemic adverse events (including fever, headache, nausea, vomiting and asthaenia), the subject was excluded from the study. After seven days of quinine treatment, the thick blood smears of all the participants proved to be negative. Thick blood smears were examined every week for 77 days or until reappearance of a patent asexual *P. falciparum *parasitaemia or loss to follow-up (missing two consecutive thick blood smears). The parasite reappearance time was defined as the number of days between the end of quinine treatment and the detection of *P. falciparum *on blood films.

Informed consent was first obtained at community level. The informed consent of each villager (or that of the parents in the case of children) was orally obtained at the beginning of the study after a thorough explanation of its purpose and was renewed at each stage of the survey. The study design received clearance from the Senegal National Ethics Committee (Dakar, Senegal) [17].

### Entomological survey

Night captures using human bait were conducted indoors and outdoors every Monday and Thursday night during the whole study period. The mosquitoes were captured from 9:00 PM until 7:00 AM by four groups of two collectors (two indoors and two outdoors), with each collector alternatively working and resting for 1 hr. The field and laboratory processing have been described previously [15].

### Statistical analysis

Differences in reappearance rate of patent *Plasmodium *parasitaemia related with epidemiological and genetic variables were tested using log-rank test (bivariate analysis) and Cox model (multivariate analysis). The BMDP 7.0 (Statistical Software, Inc., Los Angeles, CA) and SPIDA (Statistical Computing Laboratory, Eastwood, Australia) software packages were used for analysis. Due to the relative small size of the cohort, results could not be analysed for each age group, but the population was divided in two groups below or above 30 years. This cut-off value was defined by using the Akaike index for age either considered as a continuous variable or for various dichotomous variables (i.e. 1–9,1,10–19,20–30, >30, >40 years etc), and choosing the best fit [18].

## Results

One hundred eighteen persons were included and treated (114 for seven days and four for six days or less). Of the 114 persons who had received the full course of treatment, four left the village during the first week of follow-up period and were therefore excluded. A total of 110 subjects (46 M/64 F; median age [min-max]: 13.9 [1.2–72]) were monitored during the eleven weeks follow-up period and were included in the analysis. Three individuals missed two consecutive thick blood smears on the 7^th^, 42^nd ^and 57^th ^day respectively. Four individuals (3.6%) used a non-impregnated bed net. Before treatment, the prevalence of *P. falciparum *asexual blood infection was 21.4% (6/28) among subjects ≥ 30 years old versus 10.9% (9/82) in subjects < 30 years old (NS). The prevalence of patent parasitaemia did not differ according to whether an anti-malarial cure had been received during the year preceding the study or according to the time between the previous treatment and the beginning of the study.

Seven hundred forty (740) malaria vectors were captured during 128 person-night collections on human volunteers during the four-month study period, and their salivary glands examined. The resulting EIR was 15.3 infective bites per person during the 77 days follow up period.

### Reappearance time

Two individuals were infected with *Plasmodium ovale*: a 13-year old on day 71 and a 38-year old on day 76. Two other individuals were infected with *Plasmodium malariae *– two children aged 9 and 11 on day 29 and 70, respectively. The following results only concern *P. falciparum *infections.

**Figure 1:**
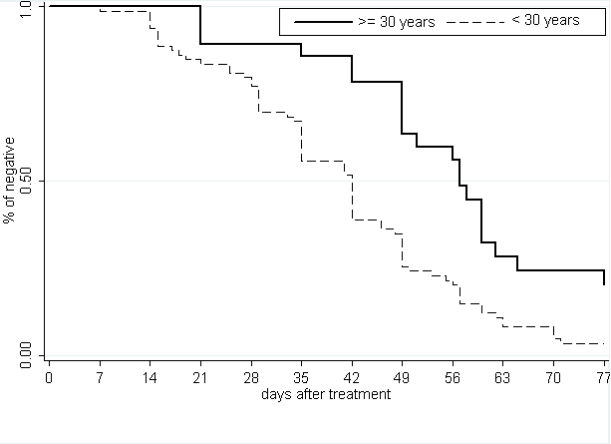
**Cumulative incidence of asexual *Plasmodium falciparum *parasitaemia as a function of time after treatment according to age group**.

The results (Figure 1 and Table 1) show that sporozoite inoculations occurring on average every five days did not each led to a patent parasitaemia. Indeed, it took more than one month to detect parasites reappearing in only 25% of the exposed population and about 50 days (corresponding to an average 10 infective bites) or only half of the population to become positive again. The median re-appearance time for the whole human population was 46.8 days. Nevertheless, at the end of the follow-up, after 77 days, the majority, 103 persons (93.6%; CI 95% [89.0–98.2]) had been re-infected with *P. falciparum*, as defined by: i) the documented clearance of parasitaemia after seven days quinine treatment and; ii) the detection of *P. falciparum *on thick blood smear during the follow-up. Among the seven persons without reappearance of *P. falciparum *blood parasitaemia, five subjects were over 30 years old. Conversely, the shortest reappearance time was seven days and was observed in an 8-years-old child.

**Table 1:** Reappearance time of asexual *P. falciparum *parasitaemia according to variables (log-rank test)

		Reappearance time of *P. falciparum *(days)			
	**n**	**25%**	**50%**	**75%**	**p-value**
**Parasitaemia before treatment**					
no	94	33.0	45.9	57.2	0.0338
yes	15	44.1	58.0	73.9	
					
**Age**					
< 30 years	82	30.7	42.7	53.5	0.0002
≥ 30 years	28	50.4	58.6	68.6	
					
**Gender**					
Female	64	33.0	48.1	58.2	0.9646
Male	46	35.0	46.4	59.8	
					
**Haemoglobin**					
AA	81	31.5	45.2	57.8	0.2486
AS	29	40.2	51.1	61.0	
					
**G6PD**					
< 6.9 μI	12	35.3	48.1	58.9	0.7743
≥ 6.9 μI	92	31.5	45.2	59.7	
					
**Roof of the hut**					
thatched	74	33.2	48.8	58.5	0.5687
zinc	36	33.6	46.5	58.9	
**Wall of the hut**					
cement	91	32.4	48.6	59.3	0.2821
banco	19	36.3	44.1	57.7	
**Free space between roof and wall**					
present	74	32.2	45.8	58.3	0.1910
absent	36	34.6	49.9	60.2	

* values are the number of days until 25 or 50 or 75% of the studied population was reinfected.

### Bivariate analysis

Bivariate analysis led to two main observations: the median reappearance time was found to be significantly longer i) in subjects having a patent *P. falciparum *parasitaemia at the time of enrolment than in parasitologically-negative individuals, and ii) in adults over 30 years as compared to younger subjects (58 days vs. 45.9; p = 0.03 and 58.6 days vs. 42.7; p = 0.0002 respectively) (Figure 1). Importantly, other parameters, such as the type of housing, the gender and the G6PD deficiency, were not associated with the time to re-infection, and individuals with the sickle cell trait demonstrated a non-significant trend to an extended time to reappearance (Table 1).

### Multivariate analysis

A multivariate Cox proportional hazards model including age, prior parasitaemia, sickle cell trait, space between the roof and the wall of the house revealed that only age ≥ 30 years and parasitaemia prior to treatment were independently associated with partial protection from reinfection. The Cox PH was 0.5 [95% CI: 0.3–0.9] for age (age < 30 years as reference group) and 0.4 [95% CI: 0.2–0.6] for prior parasitaemia (clinically non-patent parasitaemia at inclusion as reference group). Using different cut-of values than 30 years also led to conclude about the role of age, but in less significant manner.

## Discussion

In this medium-low transmission area, reinfection studies show a substantial resistance of individuals to sporozoite inoculations. Indeed it required an estimated average of more than six inoculations of sporozoites to positivate 25 percent of the population and more than 10 to positivate half of the cohort. However, after an average of 15 infective bites, all but seven individuals had been undergoing a successful liver schizogony, as shown by patent blood infection. Similar resistance to sporozoite inoculations has already been reported in individuals exposed to both high and low transmission conditions in Senegal, such as in Dielmo, with average 200 infective bites/person/year [19] and in Diohine, with 10 infective bites/person/year [20]. In Dielmo, the median times before reappearance of *P. falciparum *were 22, 39, and 53 days among age-groups 1–6, 7–14, and ≥ 15 years, respectively, i.e. confirming the strong effect of age, and showing long positivation delays in adults. After 98 days of follow up, corresponding to an average 35 infective bites, 12% of the population had not been yet become positive again, showing a stronger level of resistance to blood positivation than in Ndiop. In Diohine, the median time to reappearance of *P. falciparum *was 28 days in individuals < 40 years[20]. Similar studies were conducted in the villages of Sotuba (Mali) and in Saradidi (Kenya) respectively, areas of low and high transmission conditions. In Sotuba (Mali), the maximal reinfection rates were observed between 7 and 9 weeks post treatment; 60 of the 88 reinfections occurred during this time period. In these two studies, the analysis was restricted respectively to individuals less than twenty of age and in infants aged from 6 months to 6 years [21,22], whereas in the present study the number of individuals was larger and they belonged to all age groups. In Navrongo (Ghana), where malaria transmission is intense [23], the first infections were identified during week 3 of post-cure follow-up, the majority of new infections occurred during weeks 5–10. Here as well, 98% had finally become positive again, however only after a long delay of five months of exposure [23]. Results obtained in the very high transmission area of Idete (Tanzania), although gathered by different methods, concur to the same conclusions[24]. The EIR of 1.6 (+ 2.1) per person, per day, were much higher than the corresponding blood infection rates. The seasonal increase in EIR (> 4/night) was not accompanied by an increase in new blood infections. Results show that 93% and 99.6% of sporozoite inoculations in low and high season respectively, were blocked, i.e. did not result in a detectable blood infection, showing the acquisition of an extremely strong pre-erythrocytic or/and early erythrocytic "brake" under high transmission conditions.

The reappearance time was longer in subjects with parasitaemia upon enrolment than in individuals without, independently of age or administration of antimalarial treatment over the year preceding the study. This result is in apparent contradiction with that recorded in the higher endemic area of Dielmo, where previous parasitaemia was associated with an increased risk of the patient becoming prositive again [19]. However, this observation should be interpreted in the context of differences in anti-blood stage immunity. Indeed, in Ndiop, the clinically non-patent parasitaemia before cure, observed in only 15 individuals, was made of very low parasite densities: 70% had a parasitaemia of less than eight trophozoites per μl. In Dielmo, where transmission is continuous with higher prevalence and higher loads of parasites, the results should be analysed separately for either low or high pre-existing parasitaemia. Among the subset of individuals with only low circulating parasitsemia, the reappearance time was also longer, i.e. results are consistent with those observed in Ndiop. In the remaining, with higher initial parasite loads, the susceptibility to sporozoite inoculation increased: an increase of 1,000 trophozoites per μl was significantly associated with an increased risk of positivation by 1.6 [1.2–2.1]. The reasons for this opposite behaviour and effect upon re-positivation are not known A possible explanation is that the high parasitaemia, which are known to induce an immune suppression[25], decreased acquired defences against the sporozoite stage.

Age was significantly associated with reappearance time in all studies. However, the age at which the risk of reappearance of parasitaemia decreased significantly varied according to the studies and was grossly in relation with transmission intensity, i.e. in relation with exposure to sporozoites and hence potentially to anti-sporozoite immunity. It was significant in individuals > 40 years in Diohine [20], where the level of transmission is low, in individuals > 30 years in the present study, and in individuals > 7 years in Dielmo where the transmission is highest [19]. In Sotuba, the same age trend was observed but it was not significant most likely because of the limited age range of the study population [21]. In other words, individuals acquired faster resistance to reinfection in areas of high transmission, such as Dielmo, than in moderate areas of transmission, such as in Ndiop, or than in areas of low transmission, such as Diohine. This relationship suggests that exposure to higher numbers of sporozoites in areas of high transmission induces a stronger pre-erythrocytic immunity than in areas of moderate or low transmission, i.e. bring support to the concept of sporozoite exposure-dependant immunity. In contrast, the relative success of sporozoite inoculations is maximal in naïve non-immune individuals, as shown by blood parasitaemia induced in volunteers exposed to two infected mosquitoe bites [26], and decreases in malaria-endemic areas, as a function of EIR and age, ie of exposure to sporozoites.

Similar findings have been reported for other stages such as gametocytes, with evidence for gamete-blocking antibodies. Thus exposure to various stages of the parasite life cycle, sporozoite, liver stages, asexual blood stages, gametocytes, would generate a parasite-dependent, stage-specific immunity that modulate the survival of the corresponding stage. The lack of significant effect of the sickle cell trait upon the time it takes for a patient to become positive again may be significant in that respect.

As sickle cell trait is the strongest known factor of protection against erythrocytic stages, this observation brings support to the view that re-positivation studies measures mostly pre-erythrocytic immune defences. Indeed, in the same community, the well-documented strong effect of the sickle cell trait against clinical malaria, including uncomplicated clinical malaria, was confirmed [27], and this effect has been consistently reported elsewhere [28,29]. Thus immunity against each stage can be distinguished by distinct features. The sickle cell phenotype does not prevent blood infection, but merely reduces blood parasite densities and thereby prevents symptoms. Indeed, it is well-documented that, in hyperendemic African settings, a majority of individuals carry parasites. However pathological manifestations occur only when parasite densities in the blood are high, a phenomenon which led to define a parasite density threshold to attribute fever to malaria [30,31]. Blood stage anti-parasite immunity prevents symptoms, not infection. In contrast pre-erythrocytic stage immunity prevents infection, regardless of whether the breakthrough parasite reach, or not, a clinical patency threshold of density. Indeed in this, as in previous studies of re-positivation, the emergence of new parasites in the blood did not required treatment in the majority of cases, as pre-existing blood stage immunity controlled parasite loads below the pathogenic threshold. Together this suggests also that AS phenotype or anti-erythrocytic stages immunity modulates parasite densities, whereas pre-erythrocytic immunity modulates the emergence of new blood infections following exposure to sporozoite inoculations. Understandably both types of immunity are related to previous exposure to each stage and hence to age, as described above. The occurrence of clinical malaria corresponds to the lack of ability to control the parasite at each stage, and conversely resistance is likely dependant on the sum of these two immunities. Additional studies are required to better identify the role of each.

## Conclusion

Results obtained in this and in previous studies bring support to the existence of a resistance to sporozoite inoculation which increases, as a function of parasite transmission levels and age, i.e. depending on previous exposure to pre-erythrocytic stages. Further similar studies are required to better delineate the part related to pre-erythrocytic and to erythrocytic stages immune responses. Indeed, several malaria control measures are targeted against the pre-erythrocytic phase of the cycle, such as vector-control measures, i.e. intra-domiciliary spraying, insecticide-impregnated curtains or bed nets, repellents, and experimental vaccines directed against the sporozoite and/or liver stages, the impact of which would be best evaluated using stage-specific tools. The design and results of re-positivation studies indicate that they can contribute to monitor the efficacy of those interventions.

## Competing interests

The authors declare that they have no competing interests.

## Authors' contributions

AT responsible for field-implementation, supervision of the team in the field, data collection, analyses, writing of and finalising this paper. CS, DF contributed to entomological protocol design and data collection ABL, FDS, LM supervision of the team in the field, data collection, supported data analyses. AT supported supervision of laboratory team. RP, CR supported writing of this paper. CR established the longitudinal study of the population. PD, JFT, AS contributed to study design, overall supervision of the study, and supported data analysis and writing of this paper. All authors read and approved the final manuscript.

## Financial support

This study was supported by a grant from the Ministère Français de la Coopération et du Développement
